# Low personal exposure to benzene and 1,3-butadiene in the Swedish petroleum refinery industry

**DOI:** 10.1007/s00420-017-1234-y

**Published:** 2017-06-03

**Authors:** Pernilla Almerud, M. Akerstrom, E. M. Andersson, B. Strandberg, G. Sallsten

**Affiliations:** Department of Occupational and Environmental Medicine, Sahlgrenska University Hospital and Academy, University of Gothenburg, PO Box 414, 405 30 Gothenburg, Sweden

**Keywords:** Benzene, 1,3-butadiene, Exposure, Refinery workers, Limit of detection

## Abstract

**Purpose:**

Petroleum refinery workers are exposed to the carcinogens benzene and 1,3-butadiene. Declining exposures have been reported internationally but information on current exposure in the Swedish refinery industry is limited. The aim was to examine refinery workers’ personal exposure to benzene and 1,3-butadiene and increase awareness of exposure conditions by collaboration with involved refineries.

**Methods:**

Altogether 505 repeated personal exposure measurements were performed among workers at two refineries. Full-shift measurements were conducted in different exposure groups using Perkin Elmer diffusive samplers filled with Carbopack X. Mean levels were calculated using mixed-effects models. A large fraction of measurements below the limit of detection (LOD) required imputation of computer-generated data.

**Results:**

Mean benzene exposure among process technicians was 15.3 µg/m^3^ (95% CI 10.4–22.5 µg/m^3^) and 13.7 µg/m^3^ (95% CI 8.3–22.7 µg/m^3^) for Refinery 1 and 2, respectively. Process technicians working outdoors had higher exposure than maintenance workers (20.7 versus 5.9 µg/m^3^, *p* < 0.01). Working in the harbour and tank park (Refinery 1), compared with the process area, was associated with higher exposure. The 1,3-butadiene exposure was low, 5.4 and 1.8 µg/m^3^, respectively. The total variation was generally attributed to within-worker variability.

**Conclusions:**

Low benzene and 1,3-butadiene levels were found among refinery workers. Mean benzene exposure was about 1% of the Swedish occupational limit (1500 µg/m^3^) and for 1,3-butadiene, exposure was even lower. A large fraction of values below the LOD can be managed by carefully modelled, computer-generated data.

## Introduction

Workers in the petroleum refinery industry are exposed to benzene and 1,3-butadiene, since these substances are found in the refinery product streams (Capleton and Levy 2005; Strandberg et al. 2014; Verma and Des Tombe 1999). Benzene is a natural constituent of crude oil, which has a 0.1–3% benzene content (Verma and Des Tombe 1999). Benzene is also produced in various refining processes including catalytic reforming and catalytic dealkylation (Burns et al. 2016; Van Wijngaarden and Stewart 2003). International Agency for Research on Cancer (IARC) has reconfirmed benzene as a group 1 human carcinogen and concluded that benzene causes acute myeloid leukaemia. Also, a positive association has been observed between exposure to benzene and other subtypes of leukaemia and lymphoma (IARC 2012). The IARC also concluded that 1,3-butadiene causes cancer of the haematolymphatic organs in humans (IARC 2012).

The benzene exposure at refineries has been previously reported to cause cancer, such as leukaemia, among refinery workers (Khalade et al. 2010; Nilsson et al. 2013; Schnatter et al. 2012). Although these findings are based on work performed under historically higher benzene exposures, recent studies indicate that the leukaemia risk may be substantially greater at low exposure than previously suspected. The reason behind this is that humans metabolise benzene more efficiently at low benzene exposures compared with higher exposures (Rappaport et al. 2009).

The risk of cancer among refinery workers due to exposure to 1,3-butadiene has, to our knowledge, not been evaluated in epidemiological studies. Increased risk of leukaemia has, however, been observed in other industries such as the synthetic rubber industry (Sathiakumar et al. 2015).

Historically, high personal benzene exposures during routine work at refineries have been reported (Bates et al. 1994; Burns et al. 2016; Capleton and Levy 2005; Coker et al. 1987). However, since the mid-1980s, studies of exposure to benzene during refinery operations have indicated that full-shift exposures of refinery workers have been below 1 ppm (about 3200 µg/m^3^) (Burns et al. 2016). Technical developments and changes in the operating practices at refineries during the last decades have decreased the long-term average benzene exposure even further (Burns et al. 2016; Capleton and Levy 2005; Claydon et al. 2000). Today, the long-term average benzene exposure at refineries is expected to be low during normal operation (routine work) because most tasks performed by refinery workers are performed in proximity to a closed and continuous system. Higher exposures may be expected for work tasks involving contact with open product streams, especially in process units with a higher fraction of benzene in the product stream (Akerstrom et al. 2016; Burns et al. 2016).

There are limited data available about exposure levels in the Swedish refinery industry. A retrospective assessment of exposure to benzene was performed in a follow-up of a cohort study on petroleum refineries in Sweden (Nilsson et al. 2013) and identified the need to investigate present exposure to benzene and 1,3-butadiene in the Swedish refinery industry. Information on historical and present average 1,3-butadiene exposure during work at refineries is scarce.

The primary aim of this study was to assess average personal exposure to benzene and 1,3-butadiene and characterise the variability in exposure among workers in the petroleum refinery industry in Sweden during normal operation (randomly selected persons and days). A second aim was to develop collaboration between the researchers and refineries in order to increase knowledge about and awareness of exposure conditions.

## Materials and methods

### Study population

The majority of the Swedish petroleum refineries are situated on the west coast of Sweden and the total Swedish production of petrol (gasoline), diesel oils and heavy heating oils occurs in this area. All refineries in this area were asked to participate in a study to assess workers’ personal exposure to benzene and 1,3-butadiene. Two of the three refineries were accepted and included in the study.

Refinery 1 went into operation in 1975 and, at the time of this study (2009–2011), had about 650 employees and an annual capacity of refining about 11 million tonnes of petroleum products. The refinery consisted of a process area comprising the process units, a harbour for shipping raw oil and oil products, and tank areas. The main products produced were petrol (gasoline), diesel oils, propane, propene, butane, heavy heating oils and bunker oil.

Refinery 2 started in 1949, and in 2009–2011 had about 200 employees and an annual capacity of refining 4 million tonnes of petroleum products. The refinery consisted of a process area comprising the process units and a tank park. The main products produced were petrol (gasoline), diesel oils, heavy heating oils, liquified petroleum gas (LPG) and aviation kerosene. A minor product produced was also a benzene–toluene–xylene mixture (BTX) with a relatively high content of benzene (20%). In contrast to Refinery 1, Refinery 2 had no own harbour and their loading activities were managed by an external company in the oil harbour close to the refinery. Other products such as LPG were transported by rail.

### Measurement strategy and collaboration with the companies

The measurement strategy was designed by occupational hygienists at the University of Gothenburg and implied close cooperation with the refineries. To enable this cooperation, a study team consisting of researchers and personnel from the refineries (health and safety professionals and personnel with technical knowledge of production processes) was formed. The measurement strategy included different exposure groups consisting of refinery workers with similar estimated exposure to benzene (Table 1). The a priori assessment of the exposure was performed in close cooperation with the study team and resulted in exposure groups based mainly on a combination of occupation and work tasks. At least five workers were randomly selected from each of the a priori formed exposure groups and personal measurements were performed over a full work shift on randomly selected days. Repeated measurements were, as far as possible, performed on two sampling occasions spread over a time period of 2–5 months. This resulted in at least ten repeated measurements within each exposure group. The measurements were performed over three shifts at Refinery 1 and over two shifts at Refinery 2, with up to 15 workers (randomly selected by one of the researchers) from different exposure groups and shifts sampled during the same day. In addition, a number of samplers for “worst case” measurements (i.e. work shifts with assumed high exposures to benzene and/or 1,3-butadiene) were available at Refinery 1 upon request by the workers. Results of the worst case measurements were not included in estimations of average exposure.

**Table 1:** The *a priori* defined exposure groups at Refinery 1 and 2, description of tasks performed during a typical work shift, and the average time spent outdoors expressed as percentage of the work shift

Exposure groups	Description of tasks	Average time spent outdoors (%)^a^	
		Refinery 1	Refinery 2
Outdoor process technicians	Supervising process operations in the process area and in the harbour and tank park, working mainly outdoors. The tasks are infrequent and of short duration, except for routine work such as sampling and inspections performed on a daily basis. Also spending part of the work shift in a control room	60	51
Process area	Supervising operations in the process area, putting equipment into or taking it out of operation, taking product samples and performing minor maintenance work	60	52
Harbour and tank park	Supervising work performed in the harbour (only at Refinery 1), such as coupling and uncoupling hoses. Tasks performed in the tank park include drainage of water from tanks, taking samples and tank gauging	60	47
Indoor process technicians	Supervising process operations (in the process area and in the harbour and tank park) from a control room	7	na^b^
Outdoor maintenance workers	Performing equipment maintenance/repairs on refinery units. Workers in this group include pipe fitters, welders, instrument technicians, electricians and mechanics	41	50
Process area	Performing tasks on refinery units in the process area	37	na^b^
Harbour and tank park	Performing tasks on refinery units in the harbour and tank park	54	na^b^
Indoor maintenance workers	Performing maintenance work, mainly in indoor tool shops	20	na^b^
Laboratory workers	Performing analyses of process streams and other laboratory work including collection of samples	1	2
Engineers	Monitoring the process operations. Responsible for the design of the refinery processes and mechanical functions	9	11
Safety and emergency staff	Responsible for internal safety and emergency service, performing rescue operations, area measurements, and routine inspections and providing personal protective equipment	37	na^b^
Inspectors	Performing inspections of refinery units	31	10
Administrative personnel	Administrative and executive personnel working at the main office	11	na^b^
Railroad terminal workers	Responsible for loading products such as liquified petroleum gas (LPG) on to railroad tank cars and performing work in the tank farm	na^b^	68

^a^Information from questionnaires collected from each measured work shift

^b^Not applicable; exposure group not present/not sampled at the refinery

The results of the individual measurements were reported to the respective refinery and were shared with the workers carrying the samplers, and the initial evaluation and report were performed by the companies to increase knowledge and awareness at the company. Since the occupational exposure limit (OEL) for benzene (Swedish OEL: 1500 µg/m^3^ or 0.5 ppm) has been questioned (Akerstrom et al. 2016; Rappaport and Kupper 2008), a project-specific guideline limit of 300 µg/m^3^ was established. Results above the Swedish OELs for benzene and 1,3-butadiene (1000 µg/m^3^ or 0.5 ppm) or the project-specific guideline limit for benzene were further investigated. Although the current applicable OELs for benzene is higher in most other countries compared to Sweden (Health Council of the Netherlands 2014; Capleton and Levy 2005), this OEL still results in an unacceptable risk of leukaemia, hence the project-specific guideline value was introduced (Rappaport and Kupper 2008).

### Data collection

Personal exposure to benzene and 1,3-butadiene was measured using Perkin Elmer diffusive samplers filled with Carbopack X adsorbent. The samplers had been validated for measurements during full work shifts, both experimentally and in the refinery industry, prior to this study (Strandberg et al. 2014). Diffusive rates were 0.61 ml/min for benzene and 0.59 ml/min for 1,3-butadiene (Strandberg et al. 2014). The sampling was conducted by specifically trained employees at the respective company. The sampler was attached to the right shoulder, within the worker’s breathing zone, with the open end directed upwards. In case of rain, the sampler was provided with a protection cap and directed downwards. The personal sampling was performed during a full work shift of 8 or 12 h. The exposures were not adjusted to 8 or 12 h of sampling time; therefore, the results from the actual sampling times were used in all calculations.

After a completed shift the workers completed a questionnaire regarding work activities, time spent indoors and outdoors, and use of respiratory protective equipment when performing different tasks. The questionnaire was developed together with each refinery. In addition, weather conditions (temperature, wind direction and speed, and precipitation) were recorded on each sampling occasion.

### Chemical analysis and quality control

The analyses, performed at the Department of Occupational and Environmental Medicine, University of Gothenburg, and instrumentation are described elsewhere in detail (Strandberg et al. 2014). Briefly, the samples were analysed using a Markes Unity thermal desorber (Unity Ultra^TD^; Markes International Ltd., Llantrisant, UK) connected to an Agilent 6890 gas chromatograph (Agilent Technologies, Inc., Santa Clara, CA, USA). Controls, for the quantification and identification of target compounds, were established using two certified gas mixtures as standard reference. A calibration curve, aiming to cover the expected masses of the target compound (0.20 ng to 20 µg on the tubes), was obtained for calculating the concentrations of the analytes in the samples. Quality control (QC) samples at two pre-determined loading levels (10 and 100 ng) of benzene and 1,3-butadiene, obtained from the Dutch Metrology Institute (VSL), were analysed at the same time as the samples. The QCs did not deviate by more than 10% from the certified levels. The results from the QC samples were considered to be acceptable. Blanks were processed in parallel with the samples to assess potential residue levels of benzene and 1,3-butadiene. All samples were corrected for the blank levels. The limit of detection (LOD), calculated as three times the standard deviation of the blanks, was 5 µg/m^3^ for benzene and 1 µg/m^3^ for 1,3-butadiene.

### Statistical analysis

Data analyses were performed using SAS version 9.3 (SAS Institute, Cary, NC, USA). Statistical significance was determined at *p* < 0.05, and two-sided tests were used. Information about the within- and between-worker variability in exposure levels in each exposure group was obtained by analysing repeated exposure measurements. For a randomly selected worker and day, the personal exposure (to benzene and 1,3-butadiene) was assumed to follow a log-normal distribution according to the model:

$$\ln \left( {Xij} \right) = Yij \, = \, \mu_{Y} + \, bi \, + \, eij,$$

where *i* denotes the worker and *j* denotes the day, and where *b* and *e* are stochastic effects which are assumed to be independent and normally distributed with expected value 0 and variances $\sigma_{B}^{2}$ and $\sigma_{W}^{2}$, respectively. The total variance of *Y* in this model is $\sigma_{Y}^{2}$ = $\sigma_{B}^{2}$ +  $\sigma_{W}^{2}$.

**Fig. 1:**
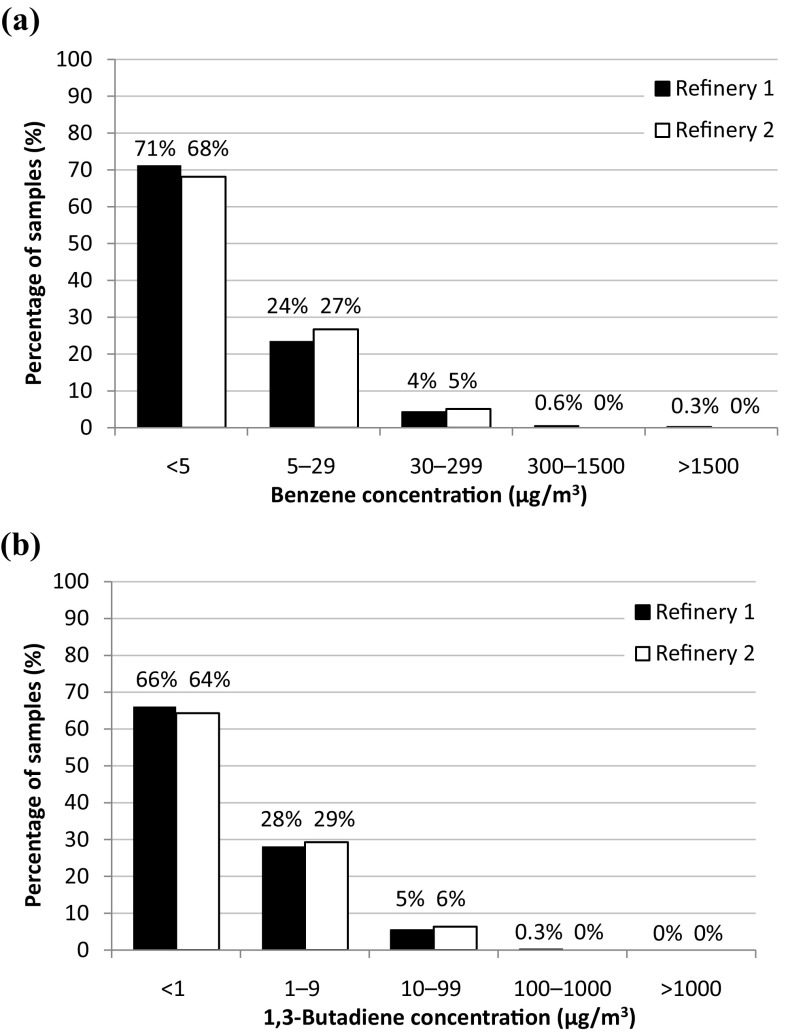
Distribution of benzene (**a**) and 1,3-butadiene (**b**) concentrations in samples from workers at Refinery 1 and 2 divided into five concentration ranges. The *first bar* represents the percentage of samples below the limit of detection (LOD) for benzene, of 5 µg/m^3^ and 1,3-butadiene, of 1 µg/m^3^

Less than half of the measurements, in total 30% for benzene and 34% for 1,3-butadiene exposures (similar fractions at both refineries), resulted in levels above the LOD (5 and 1 µg/m^3^ for benzene and 1,3-butadiene, respectively) (Fig. 1). Consequently, a special statistical method was required, otherwise the estimated average exposure would be overestimated. Jin et al. (2011) evaluated the performance of a maximum likelihood estimation for longitudinal repeated measures on log-normal data subject to left censoring (using PROC NLMIXED in SAS) and found satisfactory results for censoring levels up to 80%. Both the logarithm of the geometric mean (GM), here *μ*_*Y*_, and the within-subject variance were nearly unbiased; for the between-subject variance, the bias was less than 10%. This method was used to estimate average benzene exposure for exposure groups with more than ~15% of samples above the LOD (Table 2). For 1,3-butadiene, this method was applied to two exposure groups: process technicians and workers loading LPG (the last group at Refinery 2 only).

**Table 2:** Personal exposures to benzene (µg/m^3^) at Refinery 1 and 2

Exposure group	*n*/*N*	% >LOD	µ_X_	95% CI	Max	*µ*_*Y*_	$\sigma_{Y}^{2}$	$\sigma_{bY}^{2}$ (%)	$\sigma_{wY}^{2}$ (%)
**Refinery 1**									
Refinery process technicians	68/132	42	15.3	10.4–22.5	3775	1.6	2.2	18	82
Outdoor process technicians^a,b^	59/108	48	20.7	12.7–33.6	3775	1.7	2.6	22	78
Process area	40/71	44	9.6	6.4–15.5	144.1	1.5	1.6	9	91
Harbour and tank park	19/37	57	74.5	34.6–160.5	3775	2.2	4.1	20	80
Indoor process technicians^a^	14/24	17	3.7	3.0–4.5	7.9	1.2	0.2	19	81
Maintenance workers^b^	34/67	22	5.9	3.7–9.4	1324	0.9	1.8	14	86
Process area	21/41	20	3.6	2.7–4.9	16.7	1.0	0.6	15	85
Harbour and tank park	10/20	25	13.3	5.9–30.3	1324	1.1	3.1	11	89
Laboratory workers	13/25	32	4.6	3.4–6.2	15.4	1.3	0.4	16	84
Engineers	21/41	27	4.5	3.0–6.7	45.6	1.0	0.9	25	75
Safety and emergency staff	7/14	43	5.1	3.9–6.6	9.9	1.5	0.2	24	76
**Refinery 2**									
Outdoor process technicians	45/66	47	13.7	8.3–22.7	273.6	1.6	2.1	11	89
Process area	35/51	45	13.3	7.5–23.8	159.4	1.6	2.1	18	82
Tank park	10/15	53	15.8	5.1–48.4	273.6	1.7	2.2	3	97
Laboratory workers	6/11	45	8.4	3.4–20.7	20.0	1.7	0.9	77	23
Engineers	9/17	29	5.0	2.5–9.7	29.9	1.1	1.0	37	63

*n* number of workers, *N* number of measurements, *%* *>LOD* percentage of samples above the limit of detection (LOD), *µ*_*X*_ arithmetic mean (AM), calculated as exp(*µ*_*Y*_ + $\sigma_{Y}^{2}$/2) where *µ*_*Y*_ and $\sigma_{Y}^{2}$ are the mean and the variance of the log-transformed observations, *CI* confidence interval for the AM, *Max* maximum level detected, *µ*_*Y*_ *and* $\sigma_{Y}^{2}$ mean and variance of the log-transformed observations, $\sigma_{bY}^{2}$ *and* $\sigma_{wY}^{2}$ between- and within-individual variance of the log-transformed observations

^a^Significant difference in geometric mean (GM) between outdoor and indoor process technicians, *p* < 0.05, at Refinery 1

^b^Significant difference in GM between outdoor process technicians and maintenance workers, *p* < 0.01, at Refinery 1

Estimates of the parameters *μ*_*Y*_, $\sigma_{B}^{2}$ and $\sigma_{W}^{2}$ were obtained using PROC NLMIXED (SAS Institute), separately for each exposure group. Based on these estimated parameter values, data for imputation were computer generated (*r* = 1000 replicates) according to the model above, taking into account the correlation between measurements on the same worker. For each replicate, the observed measurements (*k*_1_ observations above the LOD) and the computer-generated data (*k*_2_ observations below the LOD) were combined and, using PROC MIXED (SAS Institute), the final estimates of the parameters (mean values and their standard errors based on 1000 replicates) were found similar to the method described in Krishnamoorthy et al. (2009). By adding a group variable to the model, *Yij* = *μ*_*Y*_ + δ·group + *bi* + *eij*, differences between exposure groups could be tested using PROC MIXED (SAS Institute) (again, the group difference was estimated from 1000 replicates). The degrees of freedom for the test were based on the number of observed measurements (on k_1_ rather than *k*_1_ + *k*_2_) and the *p* values are given as intervals (e.g. *p* < 0.05).

The arithmetic mean (AM, *μ*_*X*_) of the log-normal distribution is calculated as follows:

$${ \exp }(\mu_{\text{Y}} + \sigma_{Y}^{2} /2)$$

A confidence interval (CI) for (*μ*_*Y*_ + $\sigma_{Y}^{2}$/2) was estimated as:

$$(\hat{\mu }_{Y} + \hat{\sigma }_{Y}^{2} /2) \pm 1.96\sqrt {{\text{Var}}[\hat{\mu }_{Y} ] + (1/4){\text{Var}}[\hat{\sigma }_{Y}^{2} ]} ,$$

and the CI for AM was *e* to the power of these limits. ${\text{Var}}[\hat{\mu }_{Y} ]$ was based on the squared standard error of the intercept in the mixed model and ${\text{Var}}[\hat{\sigma }_{Y}^{2} ]$ was estimated as:

$${\text{Var}}[\hat{\sigma }_{B}^{2} ] + {\text{Var}}[\hat{\sigma }_{W}^{2} ] + 2Cov[\hat{\sigma }_{B}^{2} ,\hat{\sigma }_{W}^{2} ]$$

(the covariance matrix *Cov* for the variance estimates was produced using PROC MIXED, option ASYCOV).

Differences in exposure levels between exposure groups within each refinery were tested using the GM (GM = exp(*μ*_*Y*_)) and the *t* test of PROC MIXED in the combined dataset of observed and computer-generated data (according to Krishnamoorthy et al. 2009). For process technicians working in the process area and for laboratory workers, a corresponding comparison was also made between the two refineries.

Correlations between the benzene and 1,3-butadiene exposure were assessed using the Spearman rank correlation. The correlation was assessed using (1) only observed measurements (above LOD); and (2) the dataset combining observed and imputed computer-generated data.

In addition, ten worst case measurements among ten workers were conducted at Refinery 1 and presented separately. For calculation of average exposure among these worst case measurements, values below the LOD (two benzene samples and one 1,3-butadiene sample) were replaced by ${\text{LOD}}/\sqrt 2$ (Hornung and Reed 1990).

## Results

In total, 505 personal benzene and 1,3-butadiene samples (348 samples at Refinery 1 and 157 at Refinery 2) were collected on 265 randomly selected workers (178 and 87 at Refinery 1 and 2, respectively) from the different a priori formed exposure groups (Table 1). The measurements were carried out during 41 full work shifts in the spring and autumn of 2009 at Refinery 1, and during 34 shifts in the spring of 2010 and the autumn of 2010 and 2011 at Refinery 2. At Refinery 1, the median sampling times were 8 h (range 3.5–13 h) during weekdays and 12 h (range 10–13 h) during weekends, when the work shifts were generally longer. At Refinery 2 the median sampling time was 7 h (range 4.7–9.1 h).

The median outdoor temperature during the measurements (measured as median of the average temperature during each work shift) at Refinery 1 was 9.8 °C (range −2.0 to 21 °C) and the median wind speed was 4.7 m/s (1–14 m/s). At Refinery 2, the median temperature during measurements was 14.5 °C (8.5–17.5 °C) and the median wind speed was 5.5 m/s (2.5–10.5 m/s). Precipitation was recorded in 37 and 36% of the measured work shifts at Refinery 1 and 2, respectively.

The refinery workers spent an average of between 1 and 60% of their work shift outdoors (Table 1). Very few workers (1%) at the two refineries reported that they had used protective equipment, such as respiratory protection masks, during any task of their measured work shift.

### Personal benzene exposure in the refinery industry

The average (AM) personal benzene exposure among process technicians was 15.3 µg/m^3^ (95% CI 10.4–22.5 µg/m^3^) at Refinery 1, and 13.7 µg/m^3^ (95% CI 8.3–22.7 µg/m^3^) at Refinery 2 (Table 2). At Refinery 1, measurements were performed on both process technicians working indoors supervising the process from a control room, and process technicians working mainly outdoors. At Refinery 1 the outdoor process technicians had statistically significant higher exposure compared with indoor process technicians (AM 20.7 versus 3.7 µg/m^3^, *p* < 0.05 for the difference in GM). Among the outdoor process technicians working in the oil harbour and tank park (only Refinery 1) the benzene exposure was higher compared with those working in the process area (74.5 versus 9.6 µg/m^3^), and the difference in GM was almost statistically significant (*p* < 0.10). At Refinery 2, no significant difference was found between process technicians working in the process area and process technicians working in the tank park (Table 2).

The average personal benzene exposure of maintenance workers (only Refinery 1) was significantly lower compared with that of outdoor process technicians (5.9 versus 20.7 µg/m^3^, *p* < 0.01 for GM) (Table 2). No statistically significant difference in benzene exposure was found between maintenance workers in the process area and maintenance workers in the harbour and tank park (Table 2). Personal mean benzene exposure among laboratory workers, engineers, and safety and emergency staff (the last group only at Refinery 1) was between 4.5 and 8.4 µg/m^3^ (Table 2).

Comparisons of exposure to benzene between similar exposure groups at the two refineries (process technicians working in the process area and laboratory workers) showed no statistically significant differences.

For most of the exposure groups above, more than 50% of the total variance in benzene exposure was attributed to within-worker variability, i.e. day-to-day variability (63–97%) (Table 2). However, for laboratory workers at Refinery 2, the between-worker variability dominated (77%).

For the other occupational exposure groups—indoor maintenance workers, inspectors and administration personnel at Refinery 1 (69 samples), and maintenance workers, inspectors and railroad terminal workers at Refinery 2 (63 samples)—the average benzene exposure was low, with only a minor fraction of samples above the LOD (data not shown).

### Personal exposure to 1,3-butadiene in the refinery industry

The AM personal 1,3-butadiene exposure of process technicians was 5.4 µg/m^3^ (95% CI 3.1–9.5 µg/m^3^) and 1.8 µg/m^3^ (95% CI 1.1–2.9 µg/m^3^) at Refineries 1 and 2, respectively (Table 3). At Refinery 1, process technicians working mainly outdoors had a statistically significant higher exposure to 1,3-butadiene compared with process technicians working mainly indoors (7.2 versus 0.7 µg/m^3^, *p* < 0.05). As for benzene, process technicians working mainly outdoors in the harbour and tank park had higher exposure compared with those working in the process area (22.4 versus 3.6 µg/m^3^, *p* < 0.05) (Table 3). At Refinery 2, there was no statistically significant difference between process technicians working in the process area and process technicians working in the tank park (Table 3).

**Table 3:** Personal exposures to 1,3-butadiene (µg/m^3^) at Refinery 1 and 2

Exposure group	*n*/*N*	% >LOD	*µ*_*X*_	95% CI	Max	*µ*_*Y*_	$\sigma_{Y}^{2}$	$\sigma_{bY}^{2}$ (%)	$\sigma_{wY}^{2}$ (%)
**Refinery 1**									
Refinery process technicians	68/132	44	5.4	3.1–9.5	975.8	−0.1	3.5	16	84
Outdoor process technicians^a^	59/108	50	7.2	3.9–13.4	975.8	0.2	3.6	15	85
Process area^b^	40/71	44	3.6	1.9–6.9	79.5	−0.1	2.8	21	79
Harbour and tank park^b^	19/37	62	22.4	9.4–53.3	975.8	0.8	4.7	6	94
Indoor process technicians^a^	14/24	17	0.7	0.3–1.6	6.4	−1.2	1.8	21	79
**Refinery 2**									
Outdoor process technicians	45/66	38	1.8	1.1–2.9	90.8	−0.4	2.0	31	69
Process area	35/51	33	1.4	0.8–2.4	15.9	−0.5	1.7	47	53
Tank park	10/15	53	4.2	1.0–18.2	90.8	−0.1	3.0	11	89
Loading of LPG^c^	4/13	100	15.6	7.4–33.1	31.3	2.2	1.2	0	100

*n* number of workers, *N* number of measurements, *%* *>* *LOD* percentage of samples above the limit of detection (LOD), *µ*_*X*_ arithmetic mean (AM), calculated as exp(*µ*_*Y*_ + $\sigma_{Y}^{2}$/2) where *µ*_*Y*_ and $\sigma_{Y}^{2}$ are the mean and the variance of the log-transformed observations, *CI* confidence interval for the AM, *Max* maximum level detected, *µ*_*Y*_ *and* $\sigma_{Y}^{2}$ mean and variance of the log-transformed observations, $\sigma_{bY}^{2}$ *and* $\sigma_{wY}^{2}$ between- and within-individual variance of the log-transformed observations

^a^Significant difference in geometric mean (GM) between outdoor and indoor process technicians, *p* < 0.05

^b^Significant difference in GM between outdoor process technicians working in the process area and the harbour and tank park, *p* < 0.05

^c^Railroad terminal workers performing loading of liquified petroleum gas (LPG) on to railroad tank cars

There was no significant difference in 1,3-butadiene levels between process technicians working outdoors at the two refineries. One group (four persons) working with loading of LPG on to railroad tank cars and drainage of tanks in the tank park at Refinery 2 had an increased exposure to 1,3-butadiene compared with the rest of the exposure groups. The increased exposure was associated with the loading of LPG on to railroad tank cars, performed during 50–100% of the work time. The AM personal 1,3-butadiene exposure during these work shifts (*N* = 13 samples) was 15.6 µg/m^3^ (95% CI 7.4–33.1 µg/m^3^) (Table 3), while the exposure levels were below the LOD during work shifts when tanks were being drained (*N* = 3).

As for benzene, more than 50% of the total variance in 1,3-butadiene exposure among the process technicians was attributed to within-worker variability, i.e. day-to-day variability (53–100%) (Table 3). However, for process technicians working in the process area at Refinery 2, the within-worker variability and the between-worker variability were similar (53 and 47%, respectively).

For the other occupational exposure groups—outdoor and indoor maintenance workers, laboratory workers, engineers, inspectors, safety and emergency workers, and administration personnel at Refinery 1 (in total 216 samples), and outdoor maintenance workers, laboratory workers, engineers, and inspectors at Refinery 2 (in total 78 samples)—the average 1,3-butadiene exposure was low, with only a minor fraction of samples above the LOD (data not shown).

### Correlations between benzene and 1,3-butadiene

**Fig. 2:**
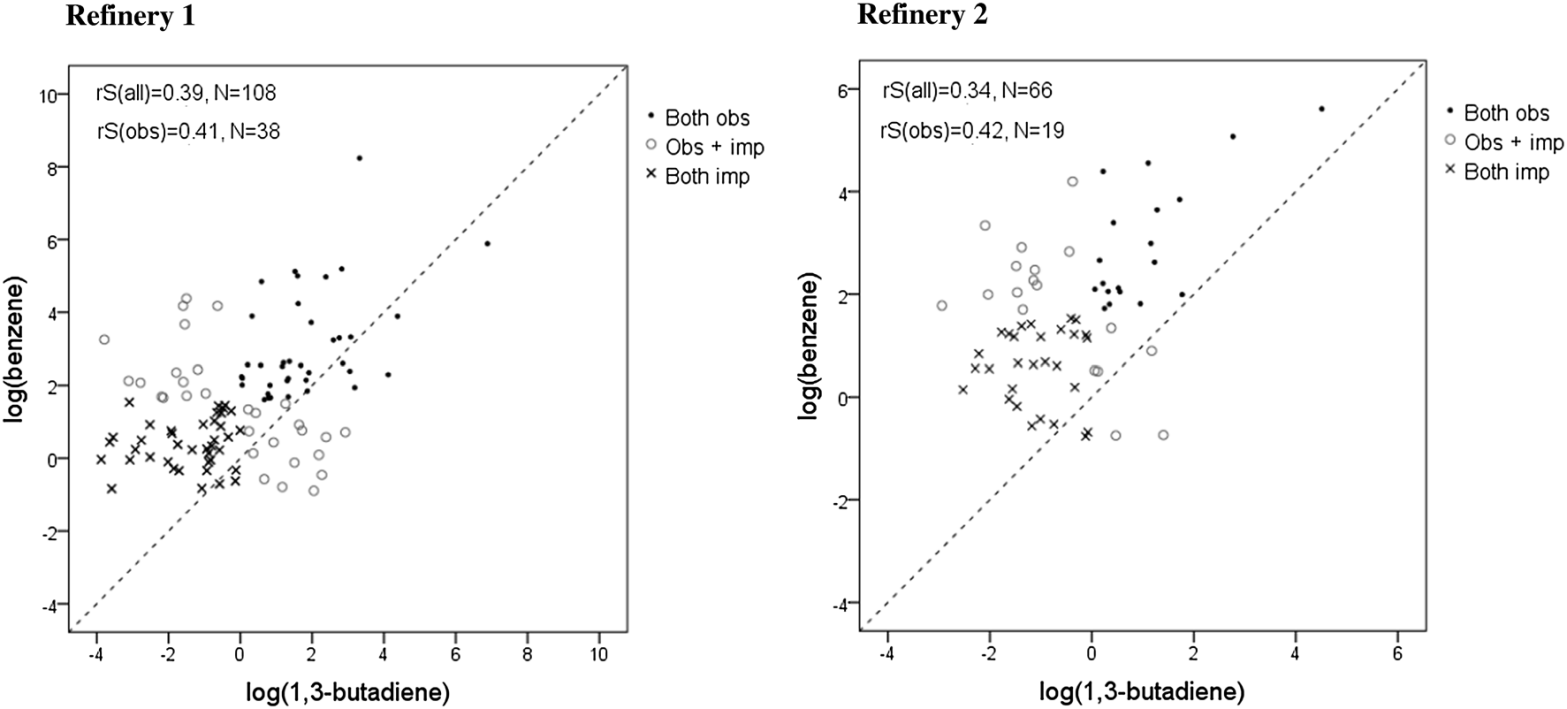
The association between benzene and 1,3-butadiene exposure in outdoor process technicians working in the process area at Refinery 1 (*left*) and Refinery 2 (*right*), with both exposures above the limit of detection (LOD) (obs) and with one or two exposures imputed using computer-generated data (imp)

Although the mean personal 1,3-butadiene exposures were ten to 100-fold lower than the benzene exposure levels measured at the same time, the two exposures were significantly correlated. The association between the benzene and the 1,3-butadiene exposure among process technicians working outdoors was estimated to be about *r*_s_ = 0.38–0.40 at both refineries (at Refinery 1, *r*_s_ = 0.39 when using all (*n* = 108) samples and *r*_s_ = 0.41 when using only samples above the LOD (*n* = 38); and at Refinery 2, *r*_s_ = 0.34 (*n* = 66) and *r*_s_ = 0.42 (*n* = 19), respectively) (Fig. 2).

### Exceedance of occupational exposure limits and the project-specific guideline limit: identified tasks with increased exposure and worst case measurements

The close cooperation between the refineries and the researchers enabled a detailed evaluation of samples exceeding the OEL and the project-specific guideline limit. In total, only one sample at Refinery 1 exceeded the OEL for benzene, of 1500 µg/m^3^, and another two samples exceeded the project-specific guideline limit of 300 µg/m^3^. The measurement exceeding the OEL (3800 µg/m^3^) was taken during normal work by a process technician in the harbour, and the two samples exceeding the project-specific guideline limit were associated with work performed by a maintenance worker and a process technician in the tank park involving drainage operations (1300 and 360 µg/m^3^, respectively). At Refinery 2, only one sample (274 µg/m^3^) was close to the project-specific guideline limit and was associated with work performed by a process technician in the tank park. Additionally, a benzene exposure of 200 µg/m^3^ was recorded in a railroad terminal worker during a work shift when loading LPG on to a railroad tank car.

No personal exposure measurements exceeded the OEL for 1,3-butadiene, of 1000 µg/m^3^, and among the process technicians, only 11% of the samples exceeded 10 µg/m^3^ (1% of the OEL). However, during drainage operations in the tank park (resulting in the benzene exposure of 360 µg/m^3^ mentioned above), a 1,3-butadiene exposure of 980 µg/m^3^ was detected.

Workers at Refinery 1 could request worst case measurements separately from the measurements based on random selection presented above. These were requested by ten workers: six process technicians working outdoors in the process area, two process technicians working outdoors in the harbour and tank park, and two maintenance workers working in the process area. In some of these cases, the sampling period, median 6.9 h (range 4.2–9.2 h), did not cover a whole work shift. The process technicians (*N* = 8) had an AM personal benzene exposure of 85.8 µg/m^3^ and a median of 19 µg/m^3^ (range 7.0–350 µg/m^3^). One measurement, of 350 µg/m^3^, exceeded the project-specific guideline value for benzene. This sample was taken on a process technician working in the process area that for a period of 30 min worked with opening and drainage of a process unit containing isomerate (a petroleum product from an isomerization reaction). This kind of work task is performed less than once a month at the refinery. The AM and median 1,3-butadiene exposure among the process technicians was 14.3 and 2.1 µg/m^3^ (range <LOD–83.4 µg/m^3^), respectively. The two maintenance workers had a benzene and 1,3-butadiene exposure below or just above the LOD.

## Discussion

This article presents average exposures to benzene and 1,3-butadiene in different exposure groups at two Swedish petroleum refineries during routine operations. Exposures during turnarounds at the same two refineries and work in a nearby oil harbour have been previously reported (Akerstrom et al. 2016). Our study approach, including full-shift personal measurements of randomly selected workers from a priori formed exposure groups measured on randomly selected work shifts, enabled us to determine average exposures, for use in epidemiological studies and for investigating compliance with OELs. In total, 505 full-shift measurements in 256 randomly selected workers were performed during 2009–2011.

The average exposures to *benzene* for all exposure groups (AM ranging from below the LOD to 75 µg/m^3^) were well below the Swedish OEL, of 1500 µg/m^3^ and only one sample exceeded this limit. Two samples exceeded the project-specific guideline limit for benzene (300 µg/m^3^). Elsewhere, much higher benzene exposures have been reported in other studies from Europe and the USA, especially in the past (Bates et al. 1994; Burns et al. 2016; Capleton and Levy 2005; Claydon et al. 2000; Coker et al. 1987; Gaffney et al. 2010, 2011 Kreider et al. 2010; Nordlinder and Ramnas 1987; Panko et al. 2009; Verma et al. 2001), with AM exposures in the range of a few hundred to thousands of µg/m^3^. The majority of the samples were below 1 ppm (about 3200 µg/m^3^), which is the OEL value applied in several countries (Capleton and Levy 2005; Health Council of the Netherlands 2014). However, the exposures reported in the literature are expected to be higher since many of the measurements reported elsewhere were conducted during worst case conditions in order to identify tasks and activities with higher potential for exposure.

A large proportion of the exposures reported in the literature were obtained during the 1980s and 1990s, when a number of operating and engineering changes were implemented. In addition, the maximum allowed content of benzene in gasoline was reduced from 5 to 1% by volume within the European Union (EU) in 2000 (Claydon et al. 2000); by comparison, in the US and Canada the benzene content has generally been below 2% on average (Verma et al. 2001). A decrease in exposures has been seen for a number of job categories in European (Claydon et al. 2000) and US refineries (Burns et al. 2016) when comparing the time periods before and after 1990. Burns et al. (2016) report a decrease from 0.27 ppm (about 860 µg/m^3^) during 1976–1989 to 0.14 ppm (about 450 µg/m^3^) during 1990–2007 when analysing samples from workers (process technicians, maintenance workers and laboratory technicians) at four US refineries during routine, startup and turnaround operations. A declining trend was also estimated for process technicians, laboratory personnel and maintenance workers at Refineries 1 and 2 in a retrospective exposure assessment, conducted as a part of a cohort study (Nilsson et al. 2013).

In the present study, the exposure group consisting of process technicians experienced the highest average benzene exposure (AM around 15 µg/m^3^) while on average the other exposure groups had about 50% or less exposure of the process technicians’ exposure. Exposure data collected from all European countries during 1993–1998 showed that maintenance workers and laboratory technicians had benzene exposures in the same range as or even higher than the process technicians, with mean levels between 220 and 410 µg/m^3^ (Claydon et al. 2000).

Work area and activities performed are important determinants of exposure (Burns et al. 2016). As expected, process technicians mainly working in a control room supervising process operations (only measured at Refinery 1) had significantly lower exposure (AM 3.7 µg/m^3^) than those working about half of their time in the process or harbour areas (AM 20.7 µg/m^3^). Moreover, outdoor process technicians working in the process areas at Refinery 1 had a lower exposure compared with process technicians working in the harbour and the tank park (9.6 versus 74.5 µg/m^3^, borderline statistically significant), probably related to more handling of open product streams in the harbour and tank park. Higher benzene exposures have previously been found among workers in the oil harbour (Akerstrom et al. 2016; Gaffney et al. 2010; Widner et al. 2011).

The exposure to *1,3*-*butadiene* among all exposure groups was low (95% of all samples were below 10 µg/m^3^) in relation to the Swedish OEL, of 1000 µg/m^3^. The exposure to 1,3-butadiene was generally considerably lower compared with the exposure to benzene, although the significant correlation obtained between the two compounds indicates a common source. 1,3-butadiene may be present in gasoline, but to a lower extent, generally below 0.1% (m/m), compared with benzene (Cecil et al. 1997).

As for benzene, the 1,3-butadiene exposures were higher for process technicians working mainly outdoors compared with those working in a control room. Also, outdoor process technicians working in the harbour and tank park had a higher exposure compared with process technicians working in the process area. Information on 1,3-butadiene exposures in the petroleum refinery industry is, to our knowledge, scarce and limited to exposure data presented in two Concawe reports (Coker et al. 1987; Claydon et al. 2000), which include measurements from all European countries. Similar to our study, the reported exposures among refinery process technicians were low, with mean values below or around 10 µg/m^3^ (Claydon et al. 2000). In contrast to our results, laboratory technicians in those reports had higher 1,3-butadiene exposure (mean 280 µg/m^3^).

Despite the low exposure to 1,3-butadiene obtained among all exposure groups in our study, railroad terminal workers responsible for loading products such as LPG on to railroad tank cars were exposed to somewhat higher levels compared with the exposure groups at the refinery. However, benzene levels were not increased for most of these workers. Liquified petroleum gas is a mixture of mainly C3 and C4 hydrocarbons, and may contain trace amounts of 1,3-butadiene (Henderson et al. 2004). Such small amounts can still be of importance regarding exposure if open handling of large amounts of LPG occurs.

Although the aim of this study was to assess the average exposure to benzene and 1,3-butadiene, using measurements from randomly selected workers and days, some tasks with higher exposures were identified in the evaluation. The single measurement exceeding the OEL for benzene (3800 µg/m^3^) was taken during work in the harbour and, according to the process technician, the vapour recovery unit was suspected of not working properly. Higher benzene exposures have previously been found among workers in the oil harbour (Akerstrom et al. 2016; Gaffney et al. 2010; Widner et al. 2011). Other identified work tasks associated with a benzene exposure close to, or exceeding, the project-specific guideline limit for benzene (300 µg/m^3^) were drainage activities, especially in the tank farm. Work tasks that involve interaction with open product streams, such as drainage of benzene-containing products and breaking and blinding operations in such units, have been found to result in higher exposure (Akerstrom et al. 2016; Burns et al. 2016). Also, increased 1,3-butadiene exposure close to the OEL was recorded during drainage activities in the tank park.

The workers at Refinery 1 had the possibility to choose to carry a sampler on any day during the measurement period if they suspected an increase in the exposure to benzene and/or 1,3-butadiene. The benzene and 1,3-butadiene exposure levels measured during these worst case measurements (*N* = 10) were somewhat higher compared with the random sampling days. One outdoor process technician had a benzene exposure of 350 µg/m^3^, exceeding the project-specific guideline value. Workers did not use respiratory protection masks in these conditions of increased exposure, and in general the use of personal protective equipment was reported to be very low.

### Cooperation with involved refineries

During the study, we worked in close cooperation with the participating refineries in order to strengthen the occupational health work at the companies. The refineries contributed their technical knowledge and experience when identifying exposure groups and evaluating data. Information obtained from the questionnaires, which were constructed in cooperation with the refineries, resulted in further improvements regarding instructions and access to correct personal protective equipment such as gloves and respiratory protection masks. A number of individuals at each refinery were trained to carry out the measurements and evaluate the results to enable collection of more samples over a prolonged sampling period, get improved feedback from the workers carrying samplers, and enable the companies to initiate monitoring programmes of their own in the future. However, the refineries did not affect the selection of workers within each exposure group or the measurement days.

### Strengths and limitations

It has been discussed whether a task-based approach should be used to assess the average benzene exposure within the downstream petroleum industry (Burns et al. 2016; Verma et al. 2001). However, full-shift measurements are needed if exposures are to be compared with OELs. Also, the area classifications at the refineries do not permit use of electric air sampling devices, which are required for short-time samplings.

In this study, repeated full-shifts measurements have been used to assess the personal exposure to benzene and 1,3-butadiene in a priori formed exposure groups. Within each exposure group, workers and days have been randomly selected and measurements of their exposure have been carried out using a passive diffusive sampler validated both in laboratory and within the industry (Strandberg et al. 2014). When conducting measurements campaigns such as this, work tasks that are rarely performed may be missed.

When using a group-based design, the conformity in exposure within the group is essential. We formed our exposure groups a priori in collaboration with experienced personnel at the refineries. The exposure groups were created based on a combination of occupation and work tasks and often coincided with work in different areas, especially for process technicians and maintenance workers, as also reported by others (Burns et al. 2016). In our study, we did not find a difference in average exposure levels between Refinery 1 and 2 for the two exposure groups process technicians working outdoors and laboratory technicians. In the present study it was not possible to compare more exposure groups due to differences in work tasks and for some exposure groups the majority of samples were below the LOD (e.g. maintenance workers). Although no measurements could be conducted on the third Swedish refinery (part of the same corporate group as Refinery 1), we expect similar exposure levels due to similar technology and start year of operation.

Generally, workers at refineries carry out a variety of tasks, some of which may involve exposure to benzene and 1,3-butadiene and some of which may be infrequently performed. As expected, the within-worker variance dominated the total variance of benzene and 1,3-butadiene exposure for all exposure groups at both refineries except for laboratory workers at Refinery 2. This may be a result of the way laboratory workers at Refinery 2 organised their work: the workers were more specialised and performed the same type of tasks over a longer time period. The exposure groups formed in our study can in most cases be regarded as homogeneous based on the criterion that between-worker variance should not exceed 20% of the total variance, which is important in testing whether a group of workers complies with the OEL (BOSH-NVvA 2011; Ogden and Lavoué 2012). However, the low exposures found in our study made such a testing procedure unnecessary. Also, the analysis of variance can provide guidance in determining whether control strategies should be targeted at the work environment (between-worker variability), or at the work conditions or practices of individual workers within a group.

### Samples below the limit of detection

A large fraction of the measurements resulted in exposures below the LOD for benzene and 1,3-butadiene, respectively. Burns et al. (2016), Gaffney et al. (2010, 2011), Kreider et al. (2010) and Panko et al. (2009) also found a high percentage of benzene levels below the LOD when performing exposure measurements in the US refinery industry. However, the LOD in general was much higher in these studies (GM 50–150 µg/m^3^, about ten to 30 times higher) compared with our study. For exposure groups with more than about 15% of their measurements above the LOD, the average personal exposure was assessed by imputation of computer-generated data, similar to the method described by Krishnamoorthy et al. (2009). Using imputation methods we decrease the risk of bias in the variance, in contrast to methods where all missing values are replaced by e.g. LOD/2. Krishnamoorthy et al. (2009) have shown that when missing values are replaced by LOD/2, the coverage of a CI for the mean can be well below the nominal value (95%).

Exposure groups with less than 15% of the values above the LOD were not possible to assess. When investigating the work tasks in these exposure groups (indoor maintenance workers, inspectors, administration personnel and railroad terminal workers), it was concluded that they had limited or no contact with open product streams and consequently had decreased benzene exposure (Akerstrom et al. 2016; Burns et al. 2016).

## Conclusions

Refinery workers in the Swedish petroleum refinery industry have a low average personal exposure to benzene and 1,3-butadiene. Mean exposures are well below the Swedish OELs, with levels of about 1% of the OEL for benzene and even lower levels for 1,3-butadiene. A large fraction of the measurements were below the LOD. We used computer-generated data to provide valid estimates of the mean personal exposure for exposure groups with more than about 15% of measurements above the LOD. The close cooperation with the refineries during this study resulted in interventions (e.g. improved instructions for use of personal protective equipment) and increased the knowledge about occupational exposure to benzene and 1,3-butadiene at the refineries. This study provides exposure data that can serve as a basis for an exposure assessment in future research on mortality and cancer incidence in the Swedish petroleum industry.
